# Measuring OPD Patient Satisfaction with Different Service Delivery Aspects at Public Hospitals in Pakistan

**DOI:** 10.3390/ijerph16132340

**Published:** 2019-07-02

**Authors:** Abid Hussain, Muhammad Asif, Arif Jameel, Jinsoo Hwang

**Affiliations:** 1 School of Public Affairs, Zijingang Campus, Zhejiang University, Hangzhou 310058, China; 2 The College of Hospitality and Tourism Management, Sejong University, 98 Gunja-Dong, Gwanjin-Gu, Seoul 143–747, Korea

**Keywords:** patient satisfaction, doctor services, nurses services, registration services, waiting time, public hospitals, Pakistan

## Abstract

The satisfaction of the patient is believed to be one of the preferred results of healthcare, and it is directly connected with the usage of health services. This study aimed to assess how doctor services, nurses’ services, and waiting time predict patient satisfaction (PS) with the service delivery of healthcare in Southern Punjab, Pakistan. The study used an exploratory research method, in which 1000 participants were selected, and used a random technique, in which 850 responses were received. Multiple regression analysis and a confirmatory factor were employed to analyze the collected data. The findings showed that doctor services (*β* = 0.232; *p* = 0.01), nurses services (*β* = 0.256; *p* = 0.01), and waiting time (*β* = 0.091; *p* = 0.03) had positive significant impacts on PS, while registration services (*β* = 0.028; *p* = 0.390) had an insignificant association with PS. Hence, a significant gap existed in the registration services that were totally ignored in hospitals of Pakistan which needed proper considerations for improvements.

## 1. Introduction

Patient satisfaction (PS) could be described as an attitude derived by a receiver of services as to whether a patient’s perceptions (expectations) for services have been fulfilled or not. The current views of the quality of service delivery seem to point out that medical care fulfills public expectancy and needs, both in regards to interpersonal care and technical care [1].

Patient satisfaction was investigated for several purposes in the healthcare delivery sector. First, it had to be decided what and how the extent of satisfaction impacts patients seeking services, fulfilling medication requirements, and their ongoing usage of these services. Satisfaction is used as an indicator of the service delivery quality as well as to help doctors and the health service institutions to build a better understanding of the patients’ feedback and to use these points of view to improve responsibility and the facilities that are provided [2].

The satisfaction of patients with health care services is a multi-aspect concept with an aspect that is connected to the main attributes of services and providers [3]. PS with medical services is considered to be of prime significance with regards to quality enhancement programs from the patients’ context, total quality management, and the anticipated results of services [4].

The healthcare sector of Pakistan is undergoing some modifications, and the application of service quality concepts to health care has a marvelous scope. Patients now have access to better health service quality in Pakistan. The most important recipients of a better healthcare system are obviously the patients and patients are of prime focus of any medical service delivery setup [5].

## 2. Theory and Hypothesis

### 2.1. Doctor Services

This feature deals with the patient’s experience concerning the service quality offered by the physicians in question. The relationship between physicians and patients involves considerable handling, which has a significant influence on the patient satisfaction level [6]. These handlings usually require proper communication guidance and information about patient problems. A very strong and recognized association is noticed between doctors and patients, that have physicians as a key preference to fulfill patients’ needs, and its assessment is mostly supported by credence aspects [7,8].

**Hypothesis 1** **(H1):** *The better the doctor services (DS), the higher the patient satisfaction.*

### 2.2. Nurses Services

Nurses services deal with patient perception about the quality of services offered by nurses during her/his stay in a hospital. Nursing care is also considered one of the most essential features in the healthcare sector. Scholars have made significant discoveries about the interaction between patient outcomes and nurses services. This literature has stressed the vigorous association between nurses services and the services delivered to the patients [9].

Using a regression model, Carman [10] measured several factors to rate the overall quality level and confirmed that the personal quality of nursing care (PQNC) with respect to health care quality was the most influencing factor of hospital care. He further validated it in another study conducted in 2000 [11].

**Hypothesis 2** **(H2):** *The better the nurses services (NS), the higher the patient satisfaction.*

### 2.3. Registration and Administrative Procedures

Hospital administrative procedures (HAP) consist of the registration process, admission, and the discharge procedure during the patient’s stay in the hospital. Moreover, Curry and Sinclair [12] stated that patients would feel less inconvenienced by their treatment if access to the health care services were improved. While providing services, the administrative delay is deemed an important aspect during the patient’s stay in the hospital at different stages.

Diaz and Ruiz [13] indicated that unreasonable delays in service provision are the cause of anger and provoke patients to react badly. Additionally, Studer [14] argued that organizations should learn from patient satisfaction to improve clinical services, collect information about staff performance, and generate ideas for the improvement and enhancement of administrative procedures and services.

**Hypothesis 3** **(H3):** *The easier the registration and administrative procedures, the higher the patient satisfaction.*

### 2.4. Waiting Time

The length of time that patients spend in hospitals to receive health services is very important. Generally, administrative procedures and medications, such as admission, discharge procedures, and waiting time for discussion and clinic appointments significantly participate in PS with the best quality of service delivery [15,16].

**Figure 1:**
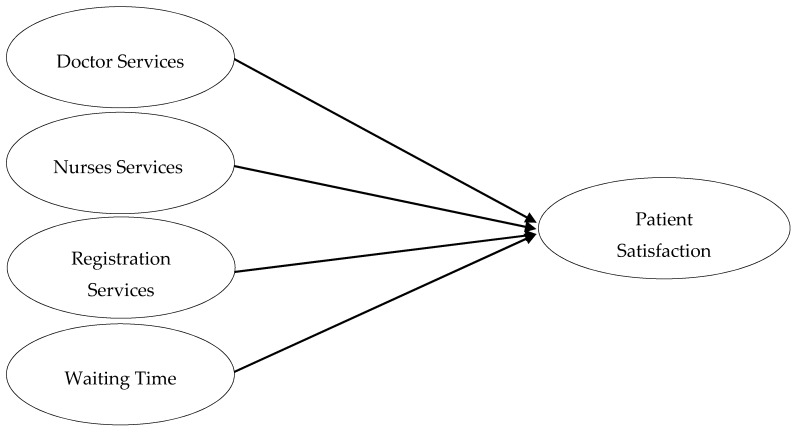
Hypothesized model.

If accessibility to health services is provided in a timely manner, then clients become less inconvenienced [12]. Unnecessary delays in facility provision have a bad impact on the process of evaluation of services which ultimately leads to patient dissatisfaction and anger [17]. Figure 1 illustrated all the hypothesized relationships.

**Hypothesis 4** **(H4):** *The shorter the waiting time, the higher the patient satisfaction.*

## 3. Materials and Methods

### 3.1. Sample

A quantitative study was designed to research the level of patient satisfaction with doctor services, nurses services, registration services, and waiting time. The study was conducted from June to August 2018 on working days, which were Monday to Saturday, in the outpatient department at public hospitals located in the Bahawalpur division, Southern Punjab Pakistan. The questionnaire was divided into two parts. The first part contained demographic information, and the second part contained the quality of medical facilities, which included the doctor services, nurses services, registration services, and waiting time. As recommended by Saunders [18] and [19], we directly invited 1000 participants, who often visit the hospitals, to participate in this study. We received 850 completed questionnaires with a response rate of 85%.

### 3.2. Instruments

The questionnaire for the present study contained five factors. The measure for PS consisted of 9 items adapted from Tucker and Adams [20]. The sample item for PS was “I have easy access to a medical specialist I need”. Doctor services (DS) included 7 items and nurses services (NS) have 8 items adapted from Xie and Or [15]. The sample items for DS and NS were “my doctors treat me in a very friendly and courteous manner” and “the nurses and assistant nurses seemed to understand how I experienced my situation”, respectively. Registration services (RS) consisted of 4 items, and waiting time (WT) contained 3 items. Both RS and WT measures were taken from Khan, et al. [21]. A sample item for RS and WT were “Medical procedures were performed correctly the first time” and “I find it hard to get an appointment for medical right away”, respectively. The demographic characteristics of the research participants included age, marital status, family income, and education (see Table 1 for more details).

We used a 5 point Likert’s scale to evaluate all items (excluding demographic details) where 5 = strongly agree and 1 = strongly disagree. For the convenience of the patients (who have no formal education) and to get good responses, the questions were orally asked in the local language (Sariki) [22].

## 4. Results

SPSS and AMOS version 25.0 were used to analyze the data. To evaluate the authenticity of all items that were studied, a valid internal reliability analysis was performed. This analysis was employed to test whether these instruments provided consistency with the results [23]. In this regard, the most commonly used technique is Cronbach’s α reliability [24]. The results of the present study demonstrated all α reliability coefficients (patient satisfaction = 0.97, doctor services = 0.94, nurses services = 0.93, registration services = 0.94, and waiting time = 0.77), which are greater than the value of 0.70 [25,26]. The means, standard deviations (SD), and correlations among all the variables and α reliabilities are illustrated in Table 2.

### 4.1. Confirmatory Factor Analysis (CFA)

A CFA was used to validate the association among the observed and the latent factors where a specific factor’s loading was assessed. Table 3 illustrates the CFA loadings of all the variables. All these values of CFA provided an adequate fit, which is evidence that they correspond to the standard criteria [26,27]. The CFA factor loadings for the present study ranged from 0.709 to 0.925, which provided a strong relationship between a variable and its underlying items. These values further confirmed the validity of all the constructs.

Moreover, the measurement model with perfect indices validated the construct validity. The factor of patient satisfaction and other factors were computed by combining all three variables in SPSS. The factors were computed by mean centering the items. Mean centering of all factors was done before running the interactional terms in the regression analysis. This process reduced multicollinearity between an interaction term and its corresponding main effects. It may have also facilitated the interpretation of regression coefficients for the interaction terms.

### 4.2. Hypotheses Testing

The results of the multiple regression analysis are illustrated in Table 4, which determined the predictors of PS in public hospitals. As can be seen in Table 4, the total variance explained by the predictors, which included doctor services, nurses services, registration services, and waiting time, and the predicted variable, such as patient satisfaction, was 37.2%. The value of Adjusted R^2^ and F value was 0.367 and 74.33, respectively, and it had a *p* = 0.001.

The range of tolerance values was 0.673 to 0.820, which is less than 0, and the range of VIF was 1.238–1.511 (a value closer to 2 illustrates the problem of collinearity), which showed that multicollinearity does not exist in data.

The values shown in Table 4 revealed that three factors (doctor services, nurses services, and waiting time) are significantly and positively affect PS. For instance, *β* value of doctor services (*β* = 0.232; t = 6.604; *p* < 0.05) showed that one unit increased in it resulted by a 0.232 unit increase with patient satisfaction, nurses services (β = 0.256; t = 7.500; *p* < 0.05) revealed that one unit increased in nurses services resulted in a 0.256 unit increase in patient satisfaction, and waiting time (β = 0.091; t = 2.884; *p* < 0.05) showed that increasing one unit in waiting time led to the decrease of 0.091 units in patient satisfaction. On the other hand, one factor was insignificant (β = 0.028; t = 0.861; *p* = 0.390). Based on these results, H1, H2, and H4 are accepted while H3 is rejected.

## 5. Discussion

Healthy people characterized by a significant fall in morbidity, mortality, and disability became the prime concern in Southern Punjab, Pakistan. This purpose can be achieved by efficient and well-managed healthcare services offered to patients to eradicate diseases [17]. The dispensing of satisfactory healthcare services is an outcome of a series of factors that reflect patients’ expectancy and experiences [28]. Our findings confirmed that services provided by doctors and nurses emerged to be significant factors that influence patient satisfaction with healthcare service delivery in Pakistan. Meanwhile, studies carried out in Norway, Iversen et al. [29], and in Iran, Zarei [30], revealed that doctor services are the significant determinant of service delivery for outpatient satisfaction. Similarly, in Pakistan, patients were also facing problems in doctor–patient relation due to less time for consultation, physical examination, discussion about health, and use of medicine [31].

Nurses services are the backbone of any healthcare system and a key determinant of patient satisfaction. In our results, we found that nurses services were a significant predictor of patient satisfaction. In the same way, Ryan and Rahman [32] also described in their study that nurses services are a significant predictor for improving patient satisfaction.

Besides the doctor and nurses services, waiting time was also exposed as an essential factor that influences patient satisfaction during service delivery. Since effective services are linked to the satisfaction, therefore, the administration tries to provide services in an efficient way. In the context of Pakistan, patients were found to face issues regarding waiting time, such as people have to wait a long time to receive examination, consultation, and medical tests [33]. Additionally, unavailability of proper cooling systems and uncomfortable seats in waiting rooms were also directly affecting patient satisfaction [34]. A study conducted by Sun et al. [35] validated that waiting time is associated with patient satisfaction improvement.

Our results revealed that registration services had an insignificant impact on patient satisfaction. Even the mean value of registration services was 2.23, which, in other words, provided the possibility of the existence of positive registration services between patients and the facilitator. In the scenario of Pakistan, patients are facing issues regarding registration services, such as no online appointment system, wards are not linked together, people have to stand for a long time to get access to each service which negatively affects patients [36]. Therefore, administration should make serious decisions to improve these services and enhance patient satisfaction with effective service delivery.

## 6. Practical Implication

Basic health is the primary need of every human. Our proposed research has the potential to help governments, concerning authorities and hospital administrations, to get rid of the increasing problems in health services in Pakistan, especially in Southern Punjab. According to the increasing population and patients’ demands, the doctors and professional nurses should be employed to facilitate patient satisfaction. The staff should be well-trained to interact carefully with the patients.

Furthermore, the registration and administrative system should be easier and less time consuming for patients. The hospital administration should focus on features such as courteous staff, queries handling at the reception counter, cooperative behavior of registration staff, and an efficient system of addressing the complaints. Our research findings thus present appropriate and related knowledge about healthcare administration that continuously provide skilled doctors and nurses with professionally pleasant handling of patients that could satisfy them and gain their loyalty. The administration can learn to provide a cooperative environment by increasing the number of doctors and nurses to decrease the waiting time of patients for obtaining services, which could also contribute towards ultimate patient satisfaction.

## 7. Limitations

This research has also some limitations. First, this study covered public hospitals in the Bahawalpur division, which has three district hospitals in Southern Punjab, Pakistan. Second, the data was only collected from the outpatient department. The findings and implication of this study cannot be generalized to other healthcare organizations or other service industries.

## 8. Conclusions

An attempt was made to evaluate the patient satisfaction level by studying the various parameters of the service delivery of OPD in district-level hospitals, which offered us specific factors that need corrective measures to enhance the further service delivery of the hospitals. The present study concludes that improving examination and consultation quality of service delivery and the information provided to the patient in the examination process, establishing or improving an internet or telephone appointment system to decrease waiting times, coordination between doctors, nurses and the outpatient department management, offering incentives for on-time doctors, generating value for the patient, and improving the doctors, nurses space of the wards can be effective strategies for the management of hospitals to increase patient satisfaction. Registration procedures should be easier in OPD so that PS could be enhanced.

## 9. Ethical Considerations

Ethical approval was obtained from the School of Public Affairs Research Ethics Committee (Zhejiang University, Hangzhou, China) and Bahawalpur Divisional Health Department ethical review board. Before data collection, all eligible respondents were informed about the aims of the study, voluntary participation, the right to withdraw at any time without giving a reason, and were assured of the confidentiality of the information to be collected.

## Figures and Tables

**Table 1:** Demographic characteristics.

Characteristics	Number	%
**Gender**		
Male	366	43.1
Female	484	56.9
**Age**		
Less than 20	69	8.1
20–29	101	11.9
30–39	178	20.9
40–49	263	30.9
50 and above	239	28.2
**Marital status**		
Married	464	54.6
Single	360	42.3
Divorced	22	2.6
Widow	4	0.5
**Education**		
No formal education	258	30.3
Primary/elementary school	277	32.6
Secondary/high school	202	23.8
College/university	45	5.3
Postgraduate	68	8.0
**Monthly income (USD)**		
Less than 1000	235	27.6
1000–1999	197	23.2
2000–2999	141	16.6
3000–3999	117	13.8
4000–4999	89	10.5
5000 or more	71	8.3

**Table 2:** Descriptive statistics, and correlations among variables.

Variable	Mean	SD	1	2	3	4	5
1. Patient Satisfaction	4.01	0.62	-				
2. Doctor Services	3.55	0.76	0.460 **	-			
3. Nurses Services	4.13	0.67	0.474 **	0.343 **	-		
4. Registration Services	2.23	0.79	0.219 **	0.208 **	0.223 **	-	
5. Waiting Time	2.85	0.82	0.258 **	0.263 **	0.157 **	0.275 **	-

Notes 1: SD = Standard Deviation; Notes 2: ** Correlation is significant at the 0.01 level (2-tailed).

**Table 3:** Results of the confirmatory factor analysis.

Construct/Factors	Items	CFA Loadings	α’s
Patient Satisfaction			0.862
	PS1	0.778	
	PS2	0.792	
	PS3	0.869	
	PS4	0.885	
	PS5	0.854	
	PS6	0.721	
	PS7	0.877	
	PS8	0.867	
	PS9	0.854	
Nurses Services			0.925
	NS1	0.762	
	NS2	0.809	
	NS3	0.788	
	NS4	0.745	
	NS5	0.793	
	NS6	0.823	
	NS7	0.804	
	NS8	0.746	
Doctor Services			0.820
	DS1	0.709	
	DS2	0.822	
	DS3	0.795	
	DS4	0.820	
	DS5	0.806	
	DS6	0.787	
	DS7	0.841	
Registration Services			0.895
	RS1	0.931	
	RS2	0.861	
	RS3	0.863	
	RS4	0.924	
Waiting Time			0.737
	WT1	0.801	
	WT2	0.810	
	WT3	0.754	

**Table 4:** Multiple regression models (dependent variable: Patient satisfaction).

Factor	Standardized Coefficients			95.0% Confidence Interval for β		Collinearity Statistics	
	St. β	T	Sig.	Lower Bound	Upper Bound	Tolerance	VIF
(Constant)		8.300	0.01	0.883	1.431		
Doctor Services	0.232 **	6.604	0.01	0.133	0.246	0.673	1.485
Nurses Services	0.256 **	7.500	0.01	0.175	0.298	0.713	1.402
Registration Services	0.028	0.861	0.390	−0.028	0.071	0.807	1.238
Waiting Time	0.091 *	2.848	0.03	0.021	0.117	0.820	1.219
Model summary		R = 0.610, R^2^ = 0.372, F = 74.33, *p* = 0.000, Durbin-Watson (DW) = 2.10					

Notes 1: Significance = ** *p* < 0.01 and * *p* < 0.05.
